# A controlled before-and-after study of a multi-modal intervention to improve hand hygiene during the peri-natal period in Cambodia

**DOI:** 10.1038/s41598-022-23937-9

**Published:** 2022-11-16

**Authors:** Yolisa Nalule, Ponnary Pors, Channa Samol, Senghort Ret, Supheap Leang, Por Ir, Alison Macintyre, Robert Dreibelbis

**Affiliations:** 1 Disease Control Department, London School of Hygiene and Tropical Medicine, London, WC1E 7HT UK; 2 WaterAid Cambodia, Phnom Penh, Cambodia; 3 National Institute of Public Health, Phnom Penh, Cambodia; 4 WaterAid Australia, Melbourne, Australia

**Keywords:** Psychology and behaviour, Neonatal sepsis, Disease prevention, Public health

## Abstract

Adequate hand hygiene practices throughout the continuum of care of maternal and newborn health are essential for infection prevention. However, the hand hygiene compliance of facility-based birth attendants, parents and other caregivers along this continuum is low and behavioural-science informed interventions targeting the range of caregivers in both the healthcare facility and home environments are scarce. We assessed the limited efficacy of a novel multimodal behaviour change intervention, delivered at the facility, to improve the hand hygiene practices among midwives and caregivers during childbirth through the return to the home environment. The 6-month intervention was implemented in 4 of 8 purposively selected facilities and included environmental restructuring, hand hygiene infrastructure provision, cues and reminders, and participatory training. In this controlled before-and-after study, the hand hygiene practices of all caregivers present along the care continuum of 99 women and newborns were directly observed. Direct observations took place during three time periods; labour, delivery and immediate aftercare in the facility delivery room, postnatal care in the facility ward and in the home environment within the first 48 h following discharge. Multilevel logistic regression models, adjusted for baseline measures, assessed differences in hand hygiene practices between intervention and control facilities. The intervention was associated with increased odds of improved practice of birth attendants during birth and newborn care in the delivery room (Adjusted odds ratio [AOR] = 4.7; 95% confidence interval [CI] = 2.7, 7.7), and that of parental and non-parental caregivers prior to newborn care in the post-natal care ward (AOR = 9.2; CI = 1.3, 66.2); however, the absolute magnitude of improvements was limited. Intervention effects were not presented for the home environment due COVID-19 related restrictions on observation duration at endline which resulted in too low observation numbers to warrant testing. Our results suggest the potential of a facility-based multimodal behaviour change intervention to improve hand hygiene practices that are critical to maternal and neonatal infection along the continuum of care.

## Introduction

Globally, infections acquired during birth and the first days of post-natal care account for an estimated 11–19% of all neonatal deaths^1^ and 10% of maternal deaths^2^, the majority of which occur in low and middle income countries (LMIC). Adequate hand hygiene practices are an essential maternal and newborn infection prevention strategy^3–6^ and should be delivered throughout the continuum of care^7–10^. The continuum of care for maternal, newborn and child health is typically defined to include care during pregnancy, delivery and postnatal care (PNC)^11,12^.

Hand hygiene compliance of birth attendants and caregivers along this care continuum remains low^13–16^. A recent systematic review of facility-based birth attendants’ hand hygiene compliance in LMIC estimated compliance ranging from 1 to 38%^13^. Observational studies of facility-based births in LMIC have identified multiple risks of infection to mothers and newborns due to inadequate hand hygiene practices during post-natal care in the facility through to the return to the household^14–19^. While global estimates are not available, both qualitative and quantitative studies suggest that hand hygiene compliance specific to newborn care is very low in low-resource settings—as low as 1% in Nigeria among healthcare workers and family members and 2.3–9.1% among mothers in Bangladesh^15,20–23^.

Hygiene interventions spanning both healthcare facility (HCF) and home environments are scarce with limited evidence around their effectiveness at changing handwashing behaviour^11,24^. Hand hygiene studies to-date mostly focus on individual care periods, particularly birth^13,17,18,25–27^ or the late post-natal period (> 7 days of life)^8,20,21,28^. Postnatal care in the critical days following facility-based birth remains under investigated^11,12^. The role and engagement of paternal and non-parental caregivers in LMIC in the provision of care across both environments has been documented^8,15,19,20,29,30^, however hand hygiene improvement strategies commonly only target birth attendants and mothers’ practices, leaving other important caregiver groups’ practices unaddressed^24^. There is a need for contextualised hand hygiene strategies that can span both the HCF and the home and effectively target hand hygiene practices of the multiple caregivers that lie along this pathway.

We tested the feasibility of a facility-based intervention targeting hand hygiene practices of caregivers of women and new-borns during institutional births and post-natal care, and the return to the home environment. For the purposes of this intervention, caregivers included midwives during childbirth, midwives, mothers, and non-maternal caregivers during post-natal care of the new-born, and mothers and other non-maternal caregivers in the home environment. Formative work in this setting documented low hand hygiene compliance in both the facilities and in the home and identified various psychosocial, physical and contextual factors influencing these practices^18,19^. Despite availability of functioning hand hygiene infrastructure and materials and demonstrated understanding of infection risk due to inadequate hand hygiene, barriers to effective hand hygiene adherence from midwives included limited understanding of hand or glove recontamination risk, habits and norms around inappropriate gloving practices, perceived lack of time from high workloads and low staffing, and low perception of newborn infection risk^18^. Among household members, barriers to effective hand hygiene included inadequate and inaccessible hygiene infrastructure in the facility, inadequate knowledge around hand hygiene opportunities and adequate hygiene protocol during newborn care, bypassing handwashing steps due to excitement or rushing to alleviate the newborn’s perceived distress (nurture), and restrictive gender norms and family hierarchies resulting in limited influence of the mother to inform other relatives’ hand hygiene practices^19^.

These findings informed the design of a multimodal behaviour change intervention delivered at facility level and aimed at improving hygiene behaviours related to maternal and neonatal care at both healthcare facilities and the home environment. The objective of this controlled before and after study was to assess the effect of this intervention on hand hygiene behaviours among birth attendants and other caregivers across eight HCFs in rural Cambodia.

## Methods

### Study setting and participants

The study was conducted in Kampong Chhnang Province, Cambodia. The study included eight purposively selected healthcare facilities (HCF), six primary health centres (PHC) and two referral hospitals, that were initially observed in the formative phase of the project^18,19^. Four HCFs were assigned to receive the intervention and the remaining four assigned to serve as comparison facilities. The six participating PHCs represented three different settings; rural/low facility-birth volume, rural/high facility-birth volume, and peri-urban/high facility-birth volume. One facility from each setting was assigned to receive the intervention. The referral hospital with the higher number of monthly deliveries was selected to receive the intervention. The sample size was considered sufficient for the exploratory nature of the study.

Any woman who presented to the HCF for delivery prior to entering the second stage of labour and was not already in excess pain and distress was eligible for recruitment. Patients considered by clinical staff to have complicated labour or delivery, or those under 18 and unaccompanied by a parent/guardian were excluded from the study. In the six PHCs, new women were recruited and enrolled over a 14-day period or until a total of five women had been enrolled; whichever came first. In the referral hospitals, new women were recruited and enrolled over a 14-day period with no limit on the total number of women enrolled per facility. Details on data collection procedures are described below.

Written consent was obtained from the women, health care workers and all accompanying members who were present with the woman at any point during the observation period. We obtained verbal consent in addition to a witness signature in the case of a participant with low literacy. Participation was voluntary and the recruitment was done in a private area and the women were encouraged to have someone else with them during the recruitment process. To minimise reactivity, the explicit mention of handwashing was avoided and participants were informed that the aim of the study was to observe care giving practices during childbirth and postnatal care. The data collector discussed and agreed verbal or non-verbal cues with each participant that they could use to pause or terminate the observation at any time.

### Intervention package

The design and development of the intervention was informed by earlier formative research conducted in the 8 HCFs^18,19^. In brief, the intervention design followed The Behaviour Centred Design (BCD) process^31^. Details on the formative research and how the BCD process was used to identify potential interventions are described in previous publications^18,19^.

Potential interventions were tested and refined through a participatory creative process using the Human-Centred Design approach led by 17 Triggers^32^, an in-country creative agency. Following a three-day co-creation workshop with key stakeholders in Phnom Penh, rapid field testing and prototyping was conducted with 15 mothers, 10 midwives, two facility directors and seven family members in two non-study HCFs and catchment communities over 3 weeks in November 2019. The final intervention design was based on the level of acceptability by users and HCF management, degree of alignment of the intervention components with formative research findings^31^ and logistic and financial constraints. The final intervention was a multimodal intervention targeting midwives, mothers, fathers and non-parental caregivers that included physical environment restructuring, provision and improved access to hand hygiene infrastructure and materials, visual cues and reminders, social influence, and participatory training.

The intervention was delivered in two locations within the maternity ward; the labour and delivery (LD) room and PNC room. The intervention components in the LD room aimed to improve hand hygiene practices of birth attendants, primarily midwives, during birth and the intervention components in the PNC room aimed to improve the hand hygiene practices of primarily mothers and caregivers providing early newborn care in both the post-natal care ward and the household following discharge. Table 1 provides an overview of the intervention components and content.

**Table 1:** Overview of intervention components and content.

Intervention location	Component	Content and purpose
Labour and delivery room	Nudges and reminders	A set of bright coloured hand hygiene icon stickers strategically placed by midwives at key points in the room where hand hygiene opportunities most often occurred during birth
		Painting the labour and delivery hand washing station area and installing a similarly coloured soap dispenser to visibly link the reminder to the required practice
	Social influence	A group commitment/pledge made by midwives to always practice proper hand hygiene and hold each other accountable
		Hand hygiene badges for midwives to wear on their uniforms meant to encourage midwives to ask each other about their hand washing activities at key points during their shifts
		A token jar and counters placed in a visible area for midwives to tally how often they reminded each other per week
	Participatory hygiene training	The training was conducted in the labour room and delivered digitally/remotely by a facilitator from the creative agency with experience in both participatory techniques and overseen by a technical lead from National Institute of Public Health—Cambodia (NIPH) and WaterAid. Midwives were trained using a combination participatory group discussions, creative exercises, scripts and role play. All training was based on Ministry of Health and WHO guidelines for technical validity
		Midwives training aimed to improve and refresh knowledge on adequate hygiene protocol specific to LD events (labour, delivery and immediate newborn care) including identifying recontamination events and the corresponding hand hygiene protocol
		With the exception of the painting, all labour and delivery room intervention components were delivered and set up by the midwives as part of the training
Post-natal care ward	Nudges and reminders	Visual demarcation of the post-natal care ward as a “clean hands” zone by painting the ward door and wall section bright green
		Placing hand hygiene icon stickers illustrating proper hand hygiene technique and nurture-evoking hygiene messaging posters across all post-natal care ward hand hygiene infrastructure and toilets
		Provision of a brightly coloured hat to each baby with visible hygiene-related messaging
	Provision of hand hygiene infrastructure and materials	Installation of a matching coloured hand washing station, liquid soap dispensers and soap at the ward entrance
		Installation of matching coloured alcohol-based handrub station at the end of each bed
		Provision of personal alcohol-based handrub bottle for each mother
	Behaviour Change Communication	Newborn hygiene—related information would be passed on to mother along with the personal alcohol-based handrub bottle and baby hat during routine care advice in the labour and delivery room following birth
		Newborn hygiene—related information would be passed on to the present visitors during the routine post-natal care advice given upon admission of the mother-newborn pair to the post-natal care ward. The ‘clean hands’ zone concept was to be explained to the visitors at this time or earlier during routine cervical checks when the admitted mother and caregivers were in PNC ward during stage 1 labour
	Participatory hygiene training	Similar to LD training component, the training was delivered digitally to the midwives via participatory group discussions, creative exercises, scripts and role play in the post-natal care ward. All training was based on National Ministry of Health and WHO guidelines for technical validity Training included: •Adequate hygiene protocol specific to newborn aftercare for all caregivers in the ward and at home •Familiarisation with the post-natal care ward intervention components including deciding how and when to introduce the intervention components and pass on the behaviour change communication to the family members and mother

The intervention was delivered to selected facilities in July 2020 and ran for 24 weeks. Throughout the 6-month implementation period, the intervention HCFs were responsible for refilling the liquid soap and maintaining the handwashing sinks. The study project team was responsible for restocking and maintaining all other intervention components. Final data collection ran from November to December 2020.

### Study design and evaluation

#### Outcomes

The study was designed as a non-randomised controlled before-and-after feasibility study. The behavioural outcomes of interest were hand hygiene practices of (1) Birth attendants during labour, delivery and immediate newborn care in the delivery room; (2) Health care workers (HCW) and other caregivers in post-natal care facility ward during newborn care and (3) Caregivers in the home environment during newborn care. Previously reported observational data on hand hygiene^18,19^ served as the baseline measures for this evaluation.

#### Data collection procedures

Data (baseline) was collected in all eight participating facilities (February–July 2019) as part of the formative research study and were repeated at the end of the implementation period (endline). All study methods, data collection procedures, tools and baseline findings are detailed in earlier publications^18,19^. The tools for structured assessments adapted from standard tools; WHO WASHFIT^33^ and Soap-Box WASH & Clean toolkit^34^ to assess the facility-level and household hygiene conditions. Hand hygiene practices were assessed via direct observation over three periods; childbirth in the delivery ward, post-natal period in the facility PNC ward and post-natal period in the home. The key events for each of the three observation periods included in the direct observation tool were selected based on previous observations of hygiene during childbirth^14^, WHO’s Five Moments for Hand hygiene^35,36^ WHO postnatal care recommendations^4,37^ and the three moments adapted for neonatal hand hygiene in the community described by Ditai et al.^38^.

Labour and delivery observations began when the woman was admitted to the facility and the first vaginal examination occurred and terminated either after 6 h or when the woman-newborn pair was discharged from the delivery room, whichever came first. Data collectors recorded key events including birth attendants’ handwashing and gloving practices and any observed contact of the birth attendants with the mother, newborn, objects and surfaces and other individuals during the observation period.

Observations during the post-natal period in the facility began when the mother-newborn pair was transferred to the PNC room of participating facilities and were terminated after a period of four continuous hours. Home observations were conducted within 72 h following discharge from the HCF and began after the household structured assessment was completed and lasted 1 h. Home observations were only completed for women who delivered in the six PHCs. During both the PNC period at the facility and the home, newborn care practices included diaper changes, cord care, breastfeeding and general newborn handling and were recorded along with any corresponding hand hygiene practices of any individuals providing newborn or maternal care.

All women were given a 15-min break from observations every 2 h, but any of the participants could pause or terminate the observations at any time either by saying so or using agreed nonverbal cues such as waving a raised hand or putting a thumbs down.

Data were collected on tablets used pre-coded observation tools by trained data collectors. Across all observations, data collectors positioned themselves in an unobtrusive location and recorded key events of all individuals present in each respective period. Five of the six study observers had prior experience with the study protocol and data collection methods having participated in the baseline data collection. All observers received a 3-day in house refresher training of the study protocol, ethical research practices and role play sessions. All observations tools and protocols were piloted in the field prior to data collection, however due to COVID-19 risk measures, the pilots were conducted in the study referral hospitals where the midwives observed four deliveries. Study observers were paired up during the pilot period to increase interrater reliability. Given the small sample size, rather than use statistical techniques to formally test reliability, observers worked independently to gather data and the observation data was compared at the end of each delivery. Any discrepancies between the pairs were discussed with the study manager for clarification and discussion.

### Data analysis

All quantitative data was analysed using StataSE 15 (Stata Corp, College Station, TX, USA)^39^. All Qualitative notes recorded during the observations were reviewed and where applicable, recoded into quantitative data using StataSE 15^39^. Data from the home and facility level assessments were analysed descriptively and triangulated to provide context to the structured observations.

### Analysis of labour and delivery structured observations

We defined labour, delivery and newborn aftercare flows according to the analysis described in the methods by Nalule et al.^18^. For each flow, we assigned time-specific hand hygiene categories (Table 2) to each birth attendant around hand hygiene opportunities prior to the initiation of the flow and within the flow when invalidation of aseptic technique occurred. Detailed definitions and descriptions of flows and aseptic procedures used for the analysis have been previously described^18^.

**Table 2:** Hand hygiene categories used in analysis.

Observation period	Hand hygiene category	Hand hygiene action
Labour and delivery (all flows)	Adequate	Handwashing with soap and new gloves (multiple or single) worn at each hand hygiene opportunity No potential recontamination of gloved and/or washed hands observed
	Inadequate	Gloves (multiple or single) are changed without intermediate handwashing with soap
	Aseptic technique invalidated	No hand hygiene actions taken at observed hand hygiene opportunity
Post-natal care in the facility Household	Adequate	Handwashing with soap and/or use of alcohol-based handrub; Handwashing with soap and glove use for any aseptic/clean procedures
	Inadequate	Handwashing with water only; Wearing gloves without intermediate handwashing with soap; No hand hygiene actions taken at observed hand hygiene opportunity

Descriptive statistics were used to calculate the frequency and proportion of flows initiated and aseptic procedures within the flows that were conducted under each respective hand hygiene category. Results were provided by data collection rounds (endline, baseline) and study group assignment (intervention, comparison).

Multilevel logistic regression models accounting for multiple observations clustered within the same facility were used to calculate difference-in-difference (DID) estimates to assess the effect of the intervention between intervention and comparison facilities after adjusting for baseline observations. To facilitate interpretability of results, the three-level primary outcome for hand hygiene during childbirth was collapsed into a series of binary outcome measures with each measure coded either as adequate hand hygiene category = 1, or aseptic technique invalidated hand hygiene category = 1 to provide better interpretation of the results to clinicians.

The outcome measures for this analysis were:

1. Adequate hand hygiene prior to the initiation of a flow or prior to any key event related to newborn care (adequate hand hygiene category = 1; aseptic technique invalidated or inadequate hand hygiene categories = 0)
2. Adequate hand hygiene during aseptic procedures within the flow (adequate hand hygiene category = 1; aseptic technique invalidated or inadequate hand hygiene categories = 0)
3. Invalidated hand hygiene prior to the initiation of a flow (aseptic technique invalidated hand hygiene category = 1; inadequate or adequate hand hygiene categories = 0).

We ran models separately for each outcome measure. Unadjusted DID models were used to calculate odds ratios without adjustment for any potential confounding variables. The second set of DID models for outcome measures was used to calculate an adjusted odds ratio and co-variates identified a priori for adjustment: working shift time, facility type (referral hospital vs primary health facility) and professional qualification (midwife vs. Doctor + Nurse vs. Midwife intern).

### Analysis of PNC and home observation structured observations

Only hand hygiene opportunities pertaining to newborn care were analysed for the PNC and home observations. Detailed descriptions of newborn-care related hand hygiene opportunities used for this analysis are previously described^19^. Caregivers of the newborns were categorised into four groups; mothers, fathers, healthcare workers (midwives, nurses, doctors and interns) or non-parental caregivers (all other individuals observed providing care to the newborn). For each caregiver, hand hygiene actions associated with each hand hygiene opportunity were coded into two categories for the analysis; adequate and inadequate (Table 2).

Analysis procedures for PNC and the household observations differed from observations in the labour and delivery wards. At baseline, we observed no hand hygiene action in relation to a hand hygiene opportunity in either the PNC or the home environment^19^ and difference-in-difference analyses were not possible. Instead, we focus on results from endline data only. Descriptive statistics were used to calculate the frequency and proportion of hand hygiene opportunities under each respective hand hygiene category by caregiver and treatment group at endline only. For the PNC, multilevel logistic regression models; unadjusted and adjusted for working shift time, facility type (referral hospital vs. primary health facility) and professional qualification (midwife vs. Doctor + Nurse vs. Midwife intern). For home observations, the limited numbers of observed hand hygiene actions did not warrant significance testing.

### Ethics approval and consent to participate

The study was conducted according to the guidelines of the Declaration of Helsinki, and approved by the Institutional Review Board at the London School of Hygiene and Tropical Medicine (Reference 16128, 21 November 2018, 22 May 2019—Amendment & Reference 19103, 04 March 2020, 01 May 2020—Amendment) and the Cambodia National Ethics Committee for Health research (NECHR Number 13, 28/01/2019, NECHR Number 134, 24 May 2019-Amendment, NEHCR Number 131, 15 June 2020). All participants provided written informed consent prior to participation.

## Results

Baseline data were collected in February—July 2019 and endline data in November—December 2020 (Table 3).

**Table 3:** Facility and household hygiene conditions.

FACILITY	Baseline		Endline	
	Comparison (n = 4)	Intervention (n = 4)	Comparison (n = 4)	Intervention (n = 4)
**Handwashing stations in delivery room**				
Available	4 (100)	4 (100)	4 (100)	4 (100)
Soap and water/Alcohol-based handrub available	4 (100)	4 (100)	4 (100)	4 (100)
**Handwashing facilities inside PNC room**				
Available	0 (0)	1 (25)	0 (0)	4 (100)
Soap and water/Alcohol-based handrub available	0 (0)	0 (0)	0 (0)	4 (100)
**Hand hygiene posters**				
Delivery room	4 (100)	4 (100)	4 (100)	4 (100)
PNC room	0 (0)	0 (0)	1 (25)	4 (100)
**Handwashing station at toilet**				
Available	4 (100)	4 (100)	4 (100)	4 (100)
Soap and water/Alcohol-based handrub available	4 (100)	2 (50)	1 (25)	3 (75)

HOUSEHOLD	Comparison (n = 11)	Intervention (n = 11)	Comparison (n = 11)	Intervention (n = 11)
**Handwashing facility**				
Available	7 (64)	10 (91)	6 (55)	9 (82)
Soap/detergent available at site	5 (45)	6 (54)	1 (9)	8 (73)
**Hand hygiene items**				
Soap available	11 (100)	10 (91)	11 (100)	9 (82)
Detergent available	11 (100)	10 (91)	10 (91)	10 (91)
Alcohol-based handrub available	0	0	1 (9)	9 (92)
**Location of alcohol-based handrub**				
Near woman	n/a	n/a	0 (0)	7 (64)
In kitchen			0 (0)	1 (9)
With household member			1 (9)	1 (9)

### Facility and household hygiene conditions

The delivery room hygiene conditions were similar between study groups at both baseline and endline (Table 3).

All delivery rooms had functional handwashing facilities (HWF). In the PNC room, none of the facilities had functional HWF or hand hygiene messaging at baseline. At endline, functional HWF were available in 100% (4/4) of the PNC rooms of the intervention facilities and 0% of comparison facilities. Hand hygiene messaging was present in 100% (4/4) of intervention and 25% (1/4) of comparison facilities.

At baseline, functioning HWF were present in 45% (5/11) of the households of the women who had given birth at the comparison facilities (comparison households) and 54% (6/11) of the households of the women who had given birth at the intervention facilities (intervention households). The presence of functioning HWF dropped to 9% (1/11) in the comparison households and increased to 73% (8/11) in the intervention households at endline. Over 90% of the households in both groups had soap and detergent available in the household at both endline and baseline. At endline, alcohol-based hand rub (ABHR) was present was in 92% (9/11) of the intervention households and 9% (1/11) of the comparison households. All ABHR present in the intervention households was from the facility.

### Participant characteristics

At baseline, 45 mothers (n = 16 comparison; n = 29 intervention) were enrolled and observed during the labour and delivery observations, 46 (n = 17 comparison; n = 29 intervention) during facility PNC observations and 22 (n = 11 comparison; n = 11 intervention) during the household observations. These details can be found in the supplementary tables provided (see Supplementary Table S1). At endline, 54 mothers (n = 19 comparison; n = 35 intervention) were observed during the labour and delivery observations, 53 (n = 18 comparison; n = 35 intervention) during facility PNC observations and 22 (n = 11 comparison; n = 11 intervention) during the household observations.

Baseline and endline characteristics of the mothers were similar between study facilities although there were some differences in working shift times at endline, number of PNC visitors at baseline and HCF staff presence in both time periods. More details showing the baseline and endline characteristics by intervention group and observation period can be found in the supplementary table (see Supplementary Table S1).

Only one additional participant was recruited for post-natal observations at baseline. At endline, one observation had to be terminated at endline due to newborn complications in the facility PNC ward.

### Structured observations in the delivery room

#### All flows

We observed a total of 251 (n = 88 comparison; n = 163 intervention) flows initiated under adequate hand hygiene at baseline and 375 (n = 122 comparison; n = 252 intervention) at endline.

When combined, the proportion of flows initiated under adequate hand hygiene declined by 10% (24% to 14%) in comparison facilities and increased by 11% (16% to 27%) in intervention facilities between baseline and endline (Table 4). After adjusting for baseline differences and potential confounders, this corresponds to an adjusted odds ratio (AOR) of 4.7 (95% confidence interval CI = 2.9, 7.7).

**Table 4:** Effect of the intervention on adequate hand hygiene during labour and delivery.

	Baseline N (%)	Endline N (%)	Percentage point difference (%)	OR^1^ (95% CI)	AOR^2^ (95% CI)
**All flows**^**3**^					
Intervention	26 (16)	69 (27)	+ 11	**3.9 (2.3, 6.4)**	**4.7 (2.9, 7.7)**
Comparison	21 (24)	17 (14)	− 10	1.00 (Ref)	1.00 (Ref)
**Labour**^**4**^					
Intervention	14 (20)	33 (45)	+ 25	**7.0 (1.4, 35.3)**	**6.6 (1.4, 32.1)**
Comparison	8 (31)	6 (17)	− 14	1.00 (Ref)	1.00 (Ref)
**Delivery**^**5**^					
Intervention	11 (17)	16 (20)	+ 3	1.8 (0.7, 5.1)	1.8 (0.5, 6.5)
Comparison	10 (26)	7 (19)	− 7	1.00 (Ref)	1.00 (Ref)
**Newborn Aftercare**^**6**^					
Intervention	2 (6)	20 (20)	+ 14	6.3 (1.0, 38.5)	**9.7 (1.2, 75.7)**
Comparison	3 (13)	4 (8)	− 5	1.00 (Ref)	1.00 (Ref)
**Aseptic events within delivery flow**^**7**^					
Intervention	26 (11)	39 (15)	+ 4	**2.4 (0.9, 5.9)**	**2.6 (0.9, 7.6)**
Comparison	21 (19)	16 (12)	− 7	1.00 (Ref)	1.00 (Ref)

Significant values are in bold.

*AOR* = adjusted odds ratio, *OR* = odds ratio.

^1^Clustered by facility.

^2^Adjusted for working shift time, facility type (referral hospital vs. primary health facility), professional qualification (midwife vs. Doctor + Nurse vs. Midwife intern).

^3^Baseline n = 251, Endline n = 375.

^4^Baseline n = 95, Endline n = 108.

^5^Baseline n = 102, Endline n = 116.

^6^Baseline n = 54, Endline n = 151.

^7^Baseline n = 340, Endline = 398.

#### Labour flow

We observed a total of 95 (n = 26 comparison; n = 69 intervention) labour flows initiated under adequate hand hygiene at baseline and 108 (n = 35 comparison; n = 73 intervention) at endline.

At endline, 45% of labour flows in intervention facilities were initiated by the healthcare worker with hands washed and clean gloves worn compared to 17% in the comparison facilities (Table 4). The proportion of labour flows initiated under adequate hygiene in the comparison facilities declined by 14% between baseline and endline and increased by 25% in the intervention facilities. After adjusting for baseline measures and potential confounders, midwives in intervention facilities had almost 7 times greater odds of practicing adequate hand hygiene prior to initiating a labour flow than midwives in comparison facilities (AOR = 6.6; CI = 1.4, 32.1).

#### Delivery flow

We observed a total of 102 (n = 39 comparison; n = 63 intervention) delivery flows initiated under adequate hand hygiene at baseline and 116 (n = 37 comparison; n = 79 intervention) at endline.

Between baseline and endline, the proportions of delivery flows initiated under adequate hand hygiene increased in the intervention facilities (17–20%) and declined in the comparison facilities (26–19%) (Table 4). We found no evidence of difference in the odds of birth attendants initiating delivery flows with adequate hand hygiene between the intervention and comparison facilities (AOR = 1.8; CI = 0.5, 6.5).

#### Hygiene within the delivery flow

Within the delivery flows, we observed 340 (n = 108 comparison; n = 232 intervention) unique aseptic events at baseline and 398 (n = 131 comparison; n = 267 intervention) at endline. The frequency and proportion of individual aseptic events in detail can be found in the supplementary information (see Supplementary Table S2). At baseline, 19% and 11% of aseptic events within the delivery flow were conducted under adequate hand hygiene in comparison and intervention facilities (Table 4). This proportion declined to 12% in comparison facilities and increased by 4% points to 15% in the intervention facilities. We found no evidence of differences in the odds of conducting any aseptic procedure within the delivery flow under adequate hygiene between the intervention and comparison group after adjusting for baseline observation and potential confounders (AOR = 2.6; CI = 0.9, 7.6). More details on the proportion of individual aseptic events conducted under adequate hand hygiene can be found in the supplementary information (see Supplementary Table S3).

#### Newborn aftercare flow

We observed a total of 54 (n = 23 comparison; n = 31 intervention) newborn aftercare flows initiated under adequate hand hygiene at baseline and 151 (n = 50 comparison; n = 101 intervention) at endline.

The proportion of newborn aftercare flows initiated under adequate hand hygiene declined from 13 to 8% in the comparison facilities and increased from 6 to 20% in intervention facilities between baseline and endline (Table 4). Midwives in an intervention facility had 10 times the odds of practicing adequate hand hygiene prior to initiating a newborn aftercare flow than midwives in the comparison facilities after adjusting for baseline observations and potential confounders (AOR = 9.7; CI = 1.2, 75.7).

#### Invalidated hand hygiene

We calculated the odds of initiating each of the flow categories under invalidated hand hygiene, coded as invalidated aseptic technique category, compared with either adequate or inadequate hand hygiene categories (Table 2).

The adjusted odds of initiating delivery flow were 70% lower (AOR = 0.3, CI = 0.1–0.7) and newborn aftercare flow 60% lower (AOR = 0.4; CI = 0.2, 0.9) for midwives in intervention facilities after adjusting for baseline observations and potential confounders**.** There was no evidence of differences in the odds of initiating the labour flow (AOR = 1.5; CI = 0.7, 3.4) with invalidated hand hygiene between intervention and comparison facilities. All details of this can be found in the supplementary information (see Supplementary Table S4).

#### Structured observations in the PNC ward

At endline, the four-hour observation period began an average of 1.6 h (range: 1–3) in comparison facilities and 1.1 h (1–3) in intervention facilities after the mother-newborn pair was discharged from the LD room (see Supplementary Table S1). The mean number of PNC visitors present during the observations was similar across the groups (4; range 1–9). In 39% (n = 7) of the observations in the comparison facilities, there was no HCW present throughout the 4-h observation period. Within the intervention facilities only, the installed sinks were functional in 94% (n = 33) of the observations and ABHR stations were functional 100% (n = 35) of the observations. 80% (n = 28) of the mothers had their personal ABHR and 86% (n = 29) of the newborns were wearing their intervention hat.

A total of 659 hand hygiene opportunities during newborn care were observed in the PNC ward at endline; 30% (n = 198) in the comparison group and 70% (n = 461) in the intervention group (Table 5).

**Table 5:** Effect of the intervention on behavioural outcomes during post-natal care in ward and household.

	Hand hygiene opportunities N (%)		Adequate hand hygiene N (%)				Odds Ratio (95% CI)^1^	Adjusted Odds ratio (95% CI)^2^
	Comparison	Intervention	Comparison	Intervention				
**Post-natal care ward**								
HCW	7 (4)	11 (2)	0 (0)		0 (0)			
Mothers	26 (13)	42 (9)	0 (0)		5 (12)			
Fathers	30 (15)	88 (19)	0 (0)		10 (11)			
Non-parental caregivers	135 (68)	320 (70)	2 (2)		27 (8)			
Total	198 (100)	461 (100)	2 (1)		42 (9)		9.8 (1.5, 63.9)^3^	9.2 (1.3, 66.2)^3^
**Home environment**								
Mothers	56 (41)	51 (41)	0 (0)			1 (2)		
Fathers	10 (7)	6 (5)	0 (0)			0 (0)		
Non-parental caregivers	71 (52)	68 (54)	0 (0)			2 (3)		
Total	137 (100)	125 (100)	0 (0)			3 (2)		

*AOR* = adjusted odds ratio, *OR* = odds ratio.

^1^Clustered by facility.

^2^Adjusted for working shift time, facility type (referral hospital vs. primary health facility), professional qualification (midwife vs. Doctor + Nurse vs. Midwife intern).

9% (42/462) of all hand hygiene opportunities were met with adequate hand hygiene practice in the intervention group by all caregiver groups except the health care workers. In the comparison group, only 1% (2/198) of all hand hygiene opportunities were conducted under adequate hand hygiene practice all of which were conducted by non-parental caregivers. After adjusting for shift time, facility type and professional qualification, caregivers in the intervention group had 9 times the odds of practicing adequate hygiene during newborn care than those in comparison group (AOR = 9.2; CI = 1.3–66.2).

### Structured observations in the home

In the home environment, the one-hour observation period took place the same day the mother-newborn pair was discharged across both study groups. The mean number of non-maternal caregivers present at home was similar and ranged from 2 to 11 in the comparison households and 3–12 in the intervention households.

A total of 262 hand hygiene opportunities were observed at home; 137 (52%) in the comparison households and 125 (48%) in the intervention households (Table 5). Non-parental caregivers accounted for over half of the hand hygiene opportunities in both comparison and intervention households. Only 2% (3/125) of the hand hygiene opportunities were conducted under adequate hand hygiene in the intervention households. No adequate hand hygiene was observed being conducted across the caregivers in the comparison households.

## Discussion

Our analyses show the potential impact of a facility-based intervention to improve hand hygiene practices among birth attendants and other caregivers during childbirth and early post-natal care in the healthcare facility environment. Compared to baseline observations and comparison facilities, birth attendants’ hand hygiene in the delivery ward improved throughout the childbirth process (all flows combined), and in particular prior to initiating maternal care during early and active labour and initiating newborn care during the 1st hour of life. The intervention was also effective in increasing adequate hand hygiene practices of mothers, fathers and non-parental caregivers prior to newborn care in the post-natal care room of the facility.

Similar to wider literature on hygiene practices in HCF^16,40–45^ and domestic settings^7,8,16,20,41^, hand hygiene practices in our context were influenced by several factors and performed by various people^18,19^. Our intervention addressed multiple identified determinants of a wide range of relevant target groups which may explain its apparent success^13,43,46–49^. Our findings are in line with multiple systematic reviews showing that interventions to improve hand hygiene behaviours are more effective when they target the context and behaviour-specific determinants of hand hygiene^46–48^. Our intervention facilitated and reinforced handwashing practice of target groups through the creation of an enabling environment (physical and social), the incorporation of several nudges & cues and leveraging the teachable moments of pregnancy/new parenthoods and facility attendance. A detailed process evaluation exploring the factors associated with the intervention success and their potential mechanisms of change is under-development.

Our intervention was successful at reducing the odds of birth attendants initiating a delivery flow with invalidated aseptic technique. However, the odds of initiating delivery flows following full hygiene protocol (hands washed with soap AND glove change) did not change. This result suggests that suboptimal hand hygiene practice, specifically glove changing without intermediate hand washing with soap, was a common occurrence in the facilities. This finding is consistent with other studies in other health care settings^36,47,50–53^ and specifically during delivery^18,40,44,45,54^ that have identified institutional factors (human resource shortages, high workload) as influencing the inadequate practice of gloving as a timesaving hand hygiene substitute. Due to limited study scope and funding, our intervention did not directly address factors at the institution level which may explain its limited effectiveness at changing gloving practices^54^.

Our findings suggest an improvement in hand hygiene compliance after events that invalidated aseptic technique, however confidence intervals overlapped the null value. Despite the persistent challenge avoiding glove/hand recontamination presents to infection control efforts in both High Income Countries (HIC)^55–57^ and LMIC^17,44,58^, its contribution to overall HCW hand hygiene compliance and subsequently nosocomial transmissions remains under investigated^17,55,59^. In labour wards in Tanzania, Gon and colleagues^17^ found that aseptic technique was invalidated almost immediately after gloving or hand hygiene action approximately half the time (227/501) A qualitative study by Hor et al.^55^ across two hospitals in Australia documented frequent risk of hand and glove recontamination during HCW team ward rounds. Further research is warranted to establish the frequency of aseptic invalidation in health care settings and understand the corresponding behavioural determinants to adapt current hand hygiene interventions to specifically address these behaviours.

Overall we observed large relative estimates of effect of the intervention among HCW and household members in the facility; however, the absolute magnitude of change was limited. Additionally, our intervention resulted in significant improvements in hand hygiene of parental and non-parental caregivers in the intervention group but the improved hand hygiene compliance was still very low (< 10%). Linking our hand hygiene intervention to the antenatal care (ANC) period may provide more opportunities to reinforce and address barriers to hand hygiene prior to childbirth. A recent systematic review found that that interventions that linked antenatal care (ANC), birth, and post-natal care were more effective in improving health outcomes compared to those with fewer linkages^12^.

Our study employed a one-time onsite training to midwives, delivered remotely over 3 days at the start of the 6-month intervention. However, multiple targeted, repetitive short on-site training sessions delivered in appropriately spaced time periods focusing on practical skill development and application have been associated with improvements in HCW knowledge and skills competency compared to the traditional concentrated one-time training approaches and has been shown to be effective in multiple LMIC context^60–63^.

Our intervention had no effect on improving hand hygiene practices in the home environment. This finding should be interpreted with caution. In alignment with the Royal Government of Cambodia’s national COVID guidelines for household data collection, the endline observation period were limited to only one hour (compared to six hours at baseline). The resulting number of observations due to this reduced observation duration period was too low for our study to sufficiently explore hygiene behaviour change across and within study groups during this critical period^64^. A longer observation period at the households would allow for sufficient data to adequately analyse our intervention effects^64^. Facility-based interventions have shown promising results as a scalable delivery platform for changing water, sanitation and hygiene (WASH) behaviours at the household level^65–68^. The HCF is hypothesized to be a uniquely placed setting to trigger behaviour change as patients and visitors are more likely to be receptive to adopting or improving health behaviours due to heightened perceptions of health risks and the benefits of prevention^69,70^. The period of pregnancy and early parenthood have also been identified as similar situations with high motivation and receptivity of those affected to improve health behaviours^70–72^. Taken together, the time spent at the facility during antenatal care, labour, delivery and post-natal care remains an opportune setting for targeting expectant parents and non-parental caregivers for sustained behaviour change at the household level^64^.

## Limitations

The measurement of behaviour could be prone to reactivity during observation. It was not possible to mask the target recipients or data collectors to the study group status of the facilities due to the visibility of the intervention. Differential reactivity between study groups could result in over- estimation of the effect of the intervention^9^. The same team of observers were used for the baseline and endline structured observations and this may have introduced observer bias during the endline data collection.

The study design, methodology and sample size limits the generalisability of our findings to the study HCFs and particularly to women with uncomplicated vaginal births in the facility. Furthermore, the non-randomisation of the facilities means that unobserved confounders may have had an effect on outcomes. We also cannot say if our intervention led to sustained adoption of the desired behaviours and a longer observation period would allow the exploration of the sustained adoption of behavioural outcomes.

As this is a limited efficacy/proof of concept study, it was not appropriate to measure maternal or newborn health related outcomes. Future larger-scale trials using measures of health impact are encouraged to determine whether the observed increases in handwashing with soap are sufficient to reduce infection.

## Conclusion

The study suggests the potential of a multi modal behaviour change intervention to improve hygiene behaviours–specifically hand hygiene- linked to maternal and neonatal infection during labour, delivery and post-natal care. More rigorous and larger scale studies are warranted to inform practice and policy change recommendations related to maternal and neonatal infection prevention.

## Supplementary Information

Supplementary Tables.

## Data Availability

The protocols, datasets used and/or analysed during this study are available from the corresponding author upon reasonable request. All data relevant to the study are included in the article or uploaded as supplementary information.

## Abbreviations

LMIC: Low and middle income countries
HCW: Health care worker
PNC: Postnatal care
LD: Labour and delivery
HCF: Healthcare facility
PHC: Primary health centres
BCD: Behaviour centred design
ABHR: Alcohol based hand rub
ANC: Antenatal care
WASH: Water, sanitation and hygiene
OR: Odds ratio
AOR: Adjusted odds ratio
CI: Confidence interval
HIC: High income country
