# Supplementary material for: A controlled before-and-after study of a multi-modal intervention to improve hand hygiene during the peri-natal period in Cambodia
Source: Sci Rep. 2022 Nov 16;12:19646. doi: 10.1038/s41598-022-23937-9 (PMC9666993; doi:10.1038/s41598-022-23937-9)
Supplement: Supplementary file 1 — Supplementary Tables. [file 41598_2022_23937_MOESM1_ESM.pdf]

SUPPLEMENTARY TABLES (S1 – S4)

A controlled before-and-after study of a multi-modal intervention to improve hand hygiene during the peri-natal period in Cambodia

Yolisa Nalule[1], Ponnary Pors[2], Channa Samol[2], Senghort Ret[2], Supheap Leang[3], Por Ir[3], Alison Macintyre[4], Robert Dreibelbis[1]

[1] Disease Control Department, London School of Hygiene and Tropical Medicine, London WC1E 7HT, UK; ynalule@gmail.com ; Robert.Dreibelbis@lshtm.ac.uk

[2] WaterAid Cambodia, Phnom Penh, Cambodia; porsponnary@gmail.com , samolchanna@yahoo.com, Senghort.ret@wateraid.org.au

[3] National Institute of Public Health, Phnom Penh, Cambodia; ipor@niph.org.kh, leangsupheap@yahoo.com

[4] WaterAid Australia, Melbourne, Australia; alison.macintyre@unimelb.edu.au

S1. Baseline and endline characteristics by intervention group and observation period

	Baseline		Endline	
**Labour and delivery room**	**Comparison (N = 16)**	**Intervention (N = 29)**	**Comparison (N = 19)**	**Intervention (N = 35)**
**Mother's age**				
Mean (SD)	27 (3.8)	28 (5.4)	29 (5.9)	30 (5.6)
Range	22 – 38	21 – 40	18 – 39	18 – 37
**Previous live births**				
Mean (SD)	2 (1.2)	2 (1.6)	2 (1.4)	1 (1.5)
Range	0 – 4	0 – 6	0 – 5	0 – 5
**HCF Travel time (mins)**				
Mean (SD)	16 (11.5)	21 (9.7)	33 (27.9)	26 (20.5)
Range	5 – 40	5 – 40	10 – 120	10 – 90
**HCF staff presence (%)**				
Primary midwife	15 (94)	16 (55)	18 (95)	17 (49)
Secondary midwife	11 (69)	22 (76)	12 (63)	28 (80)
Doctor or Nurse	1 (6)	7 (24)	1 (5)	8 (23)
Midwife intern	0 (0)	4 (14)	0 (0)	23 (66)
**Working shift time (%)**				
Morning (6.00 – 12.00)	8 (50)	12 (41)	5 (26)	16 (46)
Afternoon (12.00 – 18.00)	3(19)	6 (21)	3 (16)	6 (17)
Overnight (18.00 – 6.00)	5 (31)	11 (38)	11 (58)	13 (37)
**HCW per birth**				
Mean (SD)	3 (0.81)	2 (1.08)	2 (0.94)	3 (1.29)
Range	2 – 5	1 – 5	1 – 5	1 – 6
**Post-Natal Care**	**Comparison (N = 17)**	**Intervention (N = 29)**	**Comparison (N = 18)**	**Intervention (N = 35)**

**Time elapsed since birth (hrs)**				
Mean (SD)	1.6 (0.71)	1.4 (0.82)	1.6 (0.6)	1.1 (0.3)
Range	1 – 3	1 – 5	1 – 3	1 – 2
**Presence of HCW in PNC (%)**				
Present	17 (100)	26 (90)	11 (61)	31 (100)
Absent	0 (0)	3 (10)	7 (39)	0 (0)
**Working shift time**				
Morning (6.00 – 12.00)	5 (29)	11 (38)	6 (33)	14 (40)
Afternoon (12.00 – 18.00)	6 (35)	9 (31)	4 (22)	8 (23)
Overnight (18.00 – 6.00)	6 (35)	9 (31)	8 (44)	13 (37)
**Number of visitors in PNC**				
Mean (SD)	8 (1.7)	4 (1.8)	4 (2.0)	4 (2.1)
Range	1 – 8	2 – 8	1 – 9	2 – 8
**Intervention facilities at time of observation**				
HWF with soap and water				33 (94)
ABHR station at mother's bed				35 (100)
Mother with personal ABHR				28 (80)
Baby with intervention hat				29 (86)
**HOME**	**Comparison (N = 11)**	**Intervention (N = 11)**	**Comparison (N = 11)**	**Intervention (N = 11)**
**Days elapsed since birth**				
Mean (SD)	2 (0.75)	2 (0.47)	2 (0.54)	1 (0.4)
Range	1 - 3	1 – 2	1 – 3	1 – 3
**Days spent at home since discharge**				
Mean (SD)	0 (0.5)	0 (0.5)	0 (0.3)	0 (0.5)
Range	0 – 1	0 – 1	0 -1	0 – 1
**Presence of visitors at home**				
Mean (SD)	5 (3.2)	8 (3.3)	6 (3.3)	6 (2.8)
Range	2 – 11	3 – 13	2 – 11	3 – 12

S2. Frequency and proportion of aseptic events within delivery flow

	Baseline		Endline	
	Comparison	Intervention	Comparison	Intervention
All aseptic events (N)	108	232	131	267
Individual aseptic events % (N)				
Artificial Rupture Of Membranes	4.6 (5)	6.9 (16)	6.1 (8)	5.6 (15)
Urinary Catheter Removed	-	1.7 (4)	0.8% (1)	4.1 (11)
Episiotomy	-	3.0 (7)	3.1 (4)	3.7 (10)
Fingers in Vagina	8.3 (9)	12.5 (29)	3.8 (5)	2.2 (6)
NB face wiped during delivery	14.8 (16)	12.9 (30)	14.5 (19)	16.1 (43)
NB delivered	14.8 (16)	12.9 (30)	14.5 (19)	16.1 (43)
Cord	15.7 (17)	12.5 (29)	15.3 (20)	13.5 (36)
Placenta	14.8 (16)	15.3 (30)	15.3 (20)	13.5 (36)
VE post delivery	15.7 (17)	12.9 (30)	16.0 (21)	13.1 (35)
IV cannula- related	-	-	1.5 (2)	-
Catheter-related	-	1.7 (4)	-	1.9 (5)
Injection given	1.9 (2)	2.2 (5)	1.5 (2)	0.4 (1)
Perineum suturing	9.3 (10)	7.6 (18)	7.6 (10)	9.7 (26)

S3. Aseptic events within delivery flows conducted under adequate hand hygiene

	Baseline		Endline			
	Comparison	Intervention	Comparison	Intervention	**OR[1] (95% CI)**	**AOR[1,2] (95% CI)**
**All events % (n/N)**	19.4% (21/108)	11.2% (26/232)	12.2% (16/131)	14.6% (39/267)	2.4 (0.9 – 5.9)	2.6 (0.9 – 7.6)
Individual aseptic events % (n/N)						

Artificial Rupture Of Membranes	40.0 (2/5)	12.5 (2/16)	25.0 (2/8)	46.7 (7/15)
Urinary Catheter Removed	-	50.0 (2/4)	0.0 (0/1)	27.3 (3/11)
Episiotomy	-	14.2 (1/7)	25.0 (1/4)	30.0 (3/10)
Fingers in Vagina	22.2 (2/9)	13.8 (4/29)	0.0 (0/5)	33.3 (2/6)
NB face wiped during delivery	25.0 (4/16)	13.3 (4/30)	15.8 (3/19)	16.3 (7/43)
NB delivered	25.0 (4/16)	13.3 (4/30)	15.8 (3/19)	16.3 (7/43)
Cord	11.8 (2/17)	6.9 (4/29)	15.0 (3/20)	19.4 (7/36)
Placenta	25.0 (4/16)	10 (3/30)	5.0 (1/20)	2.8 (1/36)
VE post delivery	17.5 (3/17)	10 (3/30)	4.8 (1/21)	2.9 (1/35)
IV cannula- related	-	-	0.0 (0/2)	-
Catheter-related	-	0.0 (0/4)	-	0.0 (0/5)
Injection given	0.0 (0/2)	0.0 (0/5)	50.0 (1/2)	100 (1/1)
Perineum suturing	0.0 (0/10)	5.6 (1/18)	10 (1/10)	0.0 (0/26)

Note. AOR =Adjusted Odds Ratio, OR = Odds Ratio

1 *Clustered by facility; [2]Adjusted for facility type (referral hospital vs primary health facility), professional qualification (midwife vs Doctor + Nurse vs Midwife intern)*

S4. Effect of the intervention on invalidated hand hygiene during labour and delivery

	Baseline N (%)	Endline N (%)	% point difference (%)	OR[1] (95% CI)	AOR[2] (95% CI)
		**Invalidated hand hygiene**			
**All flows**					
Intervention	72 (45)	85 (34)	-11	0.8 (0.5, 1.2)	0.7 (0.4, 1.2)
Comparison	35 (40)	44 (36)	-4	1.00 (Ref)	1.00 (Ref)
**Labour[4]**					
Intervention	16 (23)	18 (25)	+2	1.4 (0.8, 2.5)	1.5 (0.7, 3.4)
Comparison	6 (23)	7 (20)	-3	1.00 (Ref)	1.00 (Ref)

**Delivery**[5]					
Intervention	33 (52)	27 (34)	-18	**0.4 (0.2, 0.8)**	**0.3 (0.1, 0.7)**
Comparison	14 (36)	15 (41)	+5	1.00 (Ref)	1.00 (Ref)
**Newborn Aftercare**[6]					
Intervention	24 (77)	40 (40)	-37	0.5 (0.2, 1.4)	**0.4 (0.2, 0.9)**
Comparison	15 (65)	22 (44)	-21	1.00 (Ref)	1.00 (Ref)

Note. AOR = Adjusted Odds Ratio, OR = Odds Ratio

[1] Clustered by facility; [2] Adjusted for working shift time, facility type (referral hospital vs primary health facility), professional qualification (midwife vs Doctor + Nurse vs Midwife intern); [3] Baseline n = 251, Endline n = 375; [4]Baseline n = 95, Endline n = 108; [5] Baseline n = 102, Endline n = 116; [6] Baseline n = 54, Endline n = 151; [7]Baseline n = 340, Endline = 398
